# Human Papillomavirus Genotype Landscape Across Cervical Cytology Grades and Impact of HIV Among Women of Eastern Cape Province, South Africa

**DOI:** 10.3390/v18010065

**Published:** 2025-12-31

**Authors:** Sinazo Kondlo, Nwabisa Giyose, Charles B. Businge, Zizipho Z. A. Mbulawa

**Affiliations:** 1 Department of Laboratory Medicine and Pathology, Walter Sisulu University, Mthatha 5100, South Africa; 219248966@mywsu.ac.za; 2 Department of Obstetrics and Gynaecology, Walter Sisulu University, Mthatha 5100, South Africa; nwgiyose@wsu.ac.za (N.G.); cbusinge@wsu.ac.za (C.B.B.); 3 National Health Laboratory Service, Nelson Mandela Academic Hospital, Mthatha 5100, South Africa

**Keywords:** human papillomavirus, human immunodeficiency virus, cervical cytology, HPV genotypes, cervical cancer

## Abstract

Continuous surveillance of human papillomavirus (HPV) prevalence and genotype distribution in different cervical cytology grades is necessary for cervical cancer prevention and monitoring. This study investigated the distribution of HPV genotypes and associated factors, stratified by cervical cytology grades and human immunodeficiency (HIV) status, among women in the Eastern Cape Province, South Africa. A total of 540 women were recruited from a community health facility and a referral hospital in the OR Tambo District Municipality in Eastern Cape Province. HPV detection and genotyping in cervical cells were performed using the Seegene Allplex^TM^ and Anyplex^TM^ II HPV28 assays. HPV prevalence was 60.6% among women with normal cervical cytology, 93.8% among atypical squamous cells of undetermined significance (ASC-US), 100.0% among low-grade squamous intraepithelial lesions (LSILs), 95.2% among atypical squamous cells cannot exclude high-grade lesion (ASC-H), 93.7% among high-grade squamous intraepithelial lesions (HSILs), and 92.5% among women with cervical cancer. HPV types targeted by Gardasil-9^®^ were detected in 36.0% of women with normal cervical cytology, 83.0% of those with HSIL, and 81.0% of those with cervical cancer. Among women with normal cervical cytology, HPV58, 35, and 68 were the most dominant types, HPV16, 33, and 35 in HSIL, and HPV16, 18, and 35 in cervical cancer. Differences were observed in the prevailing HPV genotype patterns when stratified by HIV infection status. This study highlights the high HPV prevalence, which further increased among women with abnormal cervical cytology. While HPV prevalence did not significantly increase with HIV co-infection, distinct differences were observed in the HPV genotype patterns when stratified by HIV status. The dominance of non-HPV vaccine types in HSIL and cervical cancer cases underscores a critical gap in current prevention strategies.

## 1. Introduction

Globally, cervical cancer remains a leading cause of cancer-related morbidity and mortality, with low-resource settings being highly affected. In South Africa, cervical cancer is the second most common cancer among women, with an estimated 11,000 new cases and over 5900 deaths annually [1,2]. In the Eastern Cape Province, prevalence and mortality remain elevated despite ongoing public health efforts [1,3]. Persistent infection with high-risk human papillomavirus (HR-HPV) genotypes is the primary cause of cervical precancerous lesions and cervical cancer [4]. Globally, HPV16 and 18 account for approximately 70% of cervical cancer cases [5]; however, HPV genotype distribution varies geographically, influencing both disease progression and vaccine efficacy [4,6].

Alphapapillomavirus are classified as either HR-HPV or low-risk HPV (LR-HPV) based on their oncogenic potential [7,8,9]. While HPV16 and 18 dominate globally in high-grade squamous intraepithelial lesion (HSIL) and cervical cancer cases, African studies report a higher burden of other HR-HPV genotypes, including HPV35, 52, 39, and 56 [10,11,12,13]. Notably, HPV35 has a high prevalence among African women, being detected in approximately 10% of cervical cancer cases in the region, compared with around 2% worldwide, which may limit the effectiveness of current HPV vaccines in Africa [12,14,15].

South Africa has implemented active cervical cancer screening and HPV vaccination programs. A national school-based HPV vaccination program was introduced in 2014, targeting girls around aged nine years in Grade 4 [16]. This program primarily delivers the Cervarix^®^ HPV vaccine, which protects against HPV16 and 18 [17,18]. However, vaccine coverage has declined since its introduction, with initial dose coverage dropping from 86% in 2014 to 37% in 2021, and second dose from 65% to 34%, partially due to the coronavirus disease 2019 (COVID-19) pandemic [19]. Commercial HPV vaccines are also available in the private sector [16]. Cervical cancer screening policy has been in place for decades, recently incorporating HR-HPV testing to triage women for liquid-based cytology. Screening officially begins at 30, except for high-risk women, such as those with HIV, who are screened at diagnosis [20,21].

The high prevalence of HIV in South Africa, affecting over 7 million people, intersects significantly with HPV-related cervical disease [22]. HIV-positive women have a higher prevalence of HPV infection, often with multiple HPV genotypes, and face increased risk of persistent HPV infection and rapid progression to invasive carcinoma compared to HIV-negative women [23,24]. HIV-induced immunosuppression impairs HPV clearance, reduces CD4+ counts, and increases viral loads, contributing to HPV persistence and lesion progression [25]. While antiretroviral therapy (ART) improves immune function, the risk of HPV-associated cervical disease remains elevated, underscoring the need for focused prevention strategies [26].

Despite the known impact of HIV on HPV infection and cervical carcinogenesis, recent data on HPV genotype distribution among South African women, particularly in rural settings, remain limited. Such data are essential to optimize screening, vaccination, and clinical management in populations with high HIV prevalence. Therefore, this study aimed to investigate the distribution of HPV genotypes and associated factors among women in the Eastern Cape Province across different cervical cytology grades and to assess the effect of HIV infection on HPV prevalence, genotype distribution, and cervical lesion severity.

## 2. Materials and Methods

### 2.1. Study Setting and Population

This cross-sectional study was conducted at a primary health facility and at Nelson Mandela Academic Hospital, both situated in the King Sabata Dalindyebo (KSD) under the OR Tambo District Municipality, in the Eastern Cape Province of South Africa. The health facility offers primary healthcare services to a diverse population residing in the surrounding areas. Nelson Mandela Academic Hospital is a large provincial, government-funded tertiary teaching hospital that serves as a regional referral center providing specialized medical services. The study population comprised women aged ≥18 years who were referred to the hospital due to abnormal cervical cytology, atypical squamous cells of undetermined significance (ASC-USs), atypical squamous cells, cannot exclude high-grade lesions (ASC-Hs), low-grade squamous intraepithelial lesions (LSILs), HSILs, and cervical cancer, and those that were attending health facility with normal cervical cytology (N = 269). Participants were recruited between June 2023 and June 2025.

The inclusion criteria included women attending the community health facility for any health-related service or referred to Nelson Mandela Academic Hospital (NMAH) for gynaecological concerns or cervical cytology results (ASCUS, ASC-H, LSIL, HSIL, or cervical cancer). Women aged 18 years and above who had previously engaged in sexual activity or were sexually active at the time of recruitment. While, exclusion criteria consisted of pregnant women, those who had never engaged in sexual activity, menstruating or bleeding due to other reasons on the day of recruitment.

### 2.2. Data Collection

Data collection involved the use of structured questionnaires to gather information on various risk factors. Participants with negative or unknown HIV status were offered an HIV rapid test. Pre and post counselling sessions were done, and a Rapid HIV test (Rapid Anti-HIV 1&2; InTec PRODUCTS, INC; Xiamen, China) was conducted by a qualified health professional. Clinical examinations of participants were conducted by an experienced medical practitioner prior to sample collection. Cervical lesion diagnoses were based on routine cytology and pathology reports; no independent morphological review was conducted for this study. Cervical specimens were collected from all participants using a Digene cervical sampler brush (Qiagen Inc., Gaithersburg, MD, USA) and immediately stored in Digene specimen transport medium (Qiagen Inc., Gaithersburg, MD, USA) and transported to Nelson Mandela Academic Hospital NHLS/WSU virology laboratory for molecular analysis. In the laboratory, cervical specimens were stored at −20 °C until the extraction of nucleic acids.

### 2.3. Laboratory Investigations

Nucleic acid extraction from cervical specimens was conducted using an automated procedure with the Seegene NIMBUS and STARMag universal extraction system, both developed by Seegene Inc. based in Seoul, Republic of Korea. The extracted nucleic acid was used for both the detection and genotyping of HPV. The Seegene Allplex^TM^ and Anyplex^TM^ II HPV28 (Seegene Inc. Seoul, Republic of Korea) multiplexed real-time type-specific polymerase chain reaction (PCR) assays were used. These assays detected, differentiated, and quantified 28 different HPV genotypes, including 13 high-risks HPV types (HPV16, 18, 31, 33, 35, 39, 45, 51, 52, 56, 58, 59, 68), 9 low-risks HPV types (HPV6, 11, 40, 42, 43, 44, 54, 61, 70), and 6 probable high-risks HPV types (HPV26, 53, 66, 69, 73, 82). Specimens from primary health facility participants were tested using the Anyplex^TM^ II HPV28 assay. While specimens from Nelson Mandela Academic Hospital-referred participants were tested using the Allplex^TM^ HPV28 assay. The current study did not compare the performance of the two assays; however, good agreement has been reported elsewhere [27,28]. The PCR assays were performed on a Bio-Rad CFX96 real-time thermocycler (Bio-Rad, Hercules, CA, USA) in accordance with the manufacturer’s instructions. The Allplex^TM^ and Anyplex^TM^ II HPV28 assays specifically targets the L1 one major capsid gene of the HPV types. Additionally, the human housekeeping (β-globin) was used as an internal control, being simultaneously with the L1 gene. Data analysis was automated and interpreted using the Seegene Viewer Software v3.31.000.006 (Seegene Inc., Seoul, Republic of Korea). The Allplex^TM^ and Anyplex^TM^ II HPV28 assays employed Seegene’s proprietary dual priming oligonucleotide and tagging oligonucleotide cleavage and extension technology in a multiplexed real-time PCR format to detect and semi-quantify HPV DNA. Viral load was categorized as high (+++; positive signal before 31 PCR cycles), medium (++; positive signal between 31 and 39 PCR cycles), or low (+; positive signal after 40 PCR cycles), with an endogenous internal control included. These viral load data are reported for completeness but were not analyzed in this study. If a sample yields a negative internal control and a negative HPV result was re-analyzed. If the re-analysis remains negative, the sample was deemed invalid. Conversely, if the internal control was negative but the HPV result was positive, the test result was considered valid. Negative HPV tests with a positive internal control were deemed negative for the 28 HPV types tested. To monitor for contamination, a negative control was included for every 20 samples and subjected to the same extraction and HPV genotyping processes.

### 2.4. Statistical Analysis

All variables from the questionnaires and laboratory investigation were captured and coded in Microsoft Excel 2016 (Microsoft Corporation, Seattle, WA, USA). GraphPad Prism v8.0.1.244 statistical software was used to perform all statistical analyses. Non-normally distributed data were summarized using the median and interquartile range (IQR). Categorical variables were presented using frequency tables, percentages, and graphs. The two-sample test of proportions was used to compare demographic characteristics between groups or categories. The association of two categorical variables was assessed using the Chi-squared test and Fisher’s exact test, depending on the expected frequencies. If 20% or more of the cells have expected frequencies of <5 or any one cell has an expected frequency of 0, Fisher’s exact test was used; otherwise, the Chi-squared test was used. The 95% Confidence Interval (CI) was used to estimate the precision of the estimate. The level of significance was set at 5% (*p*-value ≤ 0.05) for statistical significance. Risk ratios were calculated using contingency table analysis. Single HPV infection was defined as infection with one HPV type, and multiple infection was defined as infection with two or more HPV types.

### 2.5. Ethics Considerations

The study was conducted in accordance with ethical approvals granted by the Walter Sisulu University Health Research Ethics Committee (HREC: 004/2022; 037/2023; 258/2024) and the Eastern Cape Department of Health (EC_202203_011; EC_202309; EC_202502_012). Written informed consent was obtained from all participants prior to data collection, and participation was voluntary.

## 3. Results

### 3.1. Study Population Description

A total of 540 participants were included in the study, 269 had normal cytology, 16 had ASC-US, 31 had LSIL, 21 had ASC-H, 150 had HSIL and 53 had cervical cancer. The median age of overall study participants was 41 years, with an interquartile range (IQR) of 32–50 years. Among women with normal cervical cytology, most participants were aged 30–39 years (31.3%, 84/268) and were single (73.0%, 195/267). A large proportion reported no smoking (78.7%, 210/267), initiated sexual activity at 17–18 years (39.3%, 105/267), and a majority were HIV-positive (63.7%, 170/267). Among HSIL cases, the 40–49-year age group was most common (36.7%, 55/150), and about half of the participants were single (54.7%, 81/147). Most had initiated sexual activity at ≤16 years (37.7%, 55/146), and a substantial proportion were HIV-positive (72.1%, 106/147). Among women with cervical cancer, most were aged 50–98 years (60.4%, 32/53), and half were single (50.0%, 26/52). Additionally, 80.8% (42/52) reported no smoking, 67.3% (35/52) were HIV-positive, and 44.2% (23/52) had 3–4 lifetime sexual partners (Table 1).

**Figure 1:**
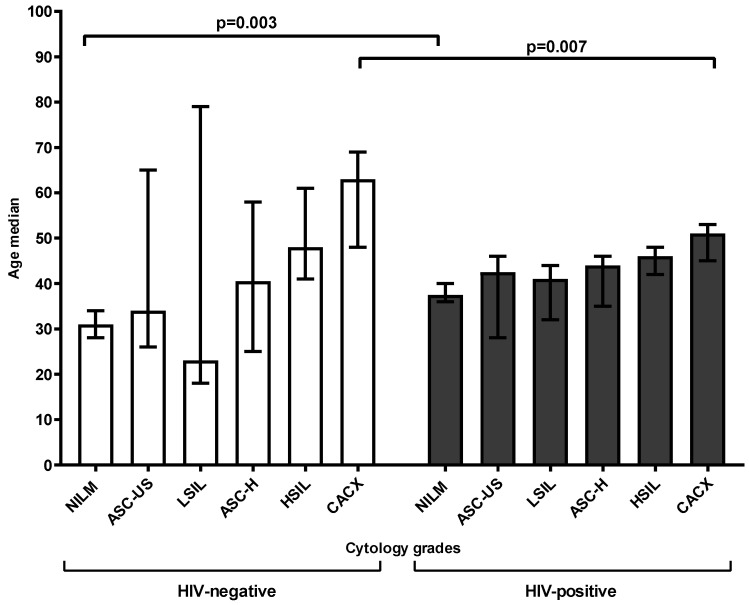
The association between HIV status, age, and cytology grades among women of Eastern Cape Province. NILM: Negative for Intraepithelial Lesion or Malignancy, ASC-US: Atypical Squamous Cells of Undetermined Significance, LSILs: Low-grade Squamous Intraepithelial Lesions, ASC-H: Atypical Squamous Cells cannot exclude High-grade Lesion, HSILs: High-grade Squamous Intraepithelial Lesions, CACX: Cervical Cancer.

Among both HIV-negative and HIV-positive women, the median age increased significantly as the cytology grades worsened (*p* for trend < 0.0001, *p* for trend < 0.001, respectively). HIV-negative women with negative intraepithelial lesions or malignancy (NILM) (median: 31 years) were younger than those with cervical cancer (median: 63 years, *p* < 0.0001). A similar age trend was observed among the HIV-positive women (*p* < 0.0001). HIV-negative women were younger in the NILM group than HIV-positive women (*p* = 0.003), while HIV-negative women were older in the cervical cancer group than HIV-positive women (*p* = 0.007, Figure 1).

### 3.2. Prevalence of HPV Infection According to Cervical Cytology and HIV Status Among Eastern Cape Women

Among women with NILM cytology, the prevalence of HPV infection was 60.6% (163/269), with multiple infections (30.9%, 83/269) being slightly more common than single infections (29.7%, 80/269). HR-HPV types (47.2%, 127/269) were also more common than LR-HPV types (27.9%, 75/269, Table 2). In contrast, women with HSIL had higher rates, with 93.7% (145/150) positive for any HPV type, 80.7% (121/150) having multiple HPV infections and 95.3% (143/150) positive for HR-HPV types. Among cervical cancer cases, these rates remained high, with 92.5% (49/53) positive for HPV infection, 64.2% (34/53) having multiple infections and 90.6% (48/53) positive for HR-HPV (Table 2). The overall HPV prevalence in different cervical cytology groups was not significantly influenced by HIV coinfection except in HSIL group (99.1%, 105/106 compared to 90.2%, 37/41; RR: 1.10, 0.99–1.22, *p* = 0.022).

**Figure 2:**
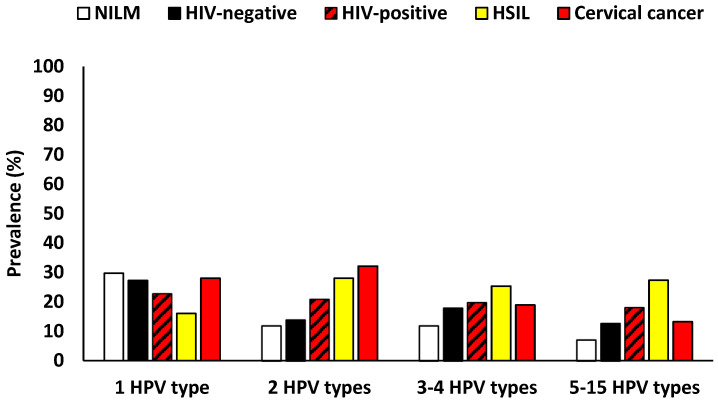
Prevalence of number of HPV types detected among Eastern Cape women. NILM: Negative for Intraepithelial Lesion or Malignancy, HSILs: High-grade Squamous Intraepithelial Lesions.

Among women with NILM cytology, infection with one HPV type (29.7%, 80/269) was more common than infection with five to fifteen HPV types (7.1%, 19/269). In HSIL cases, infection with two HPV types (28.0%, 42/150) and with five to fifteen HPV types (27.3%, 41/150) were more prevalent than infection with one HPV type (16.0%, 24/150). Among cervical cancer cases, infection with one or two HPV types (28.3%, 15/52 and 32.1%, 17/53, respectively) was more common than infection with five to fifteen HPV types (13.2%, 7/53). In HIV-negative women, infection with one HPV type (27.2%, 47/173) was more common than infection with five to fifteen HPV types (12.7%, 22/173). Similarly, in HIV-positive women, infection with one HPV type (22.7%, 82/361) was more prevalent than infection with five to fifteen HPV types (18.0%, 65/361, Figure 2).

### 3.3. Distribution of HPV Genotypes Among Women with Different Cervical Cytology Grades in Single and Multiple Infections According to HIV Status

**Figure 3:**
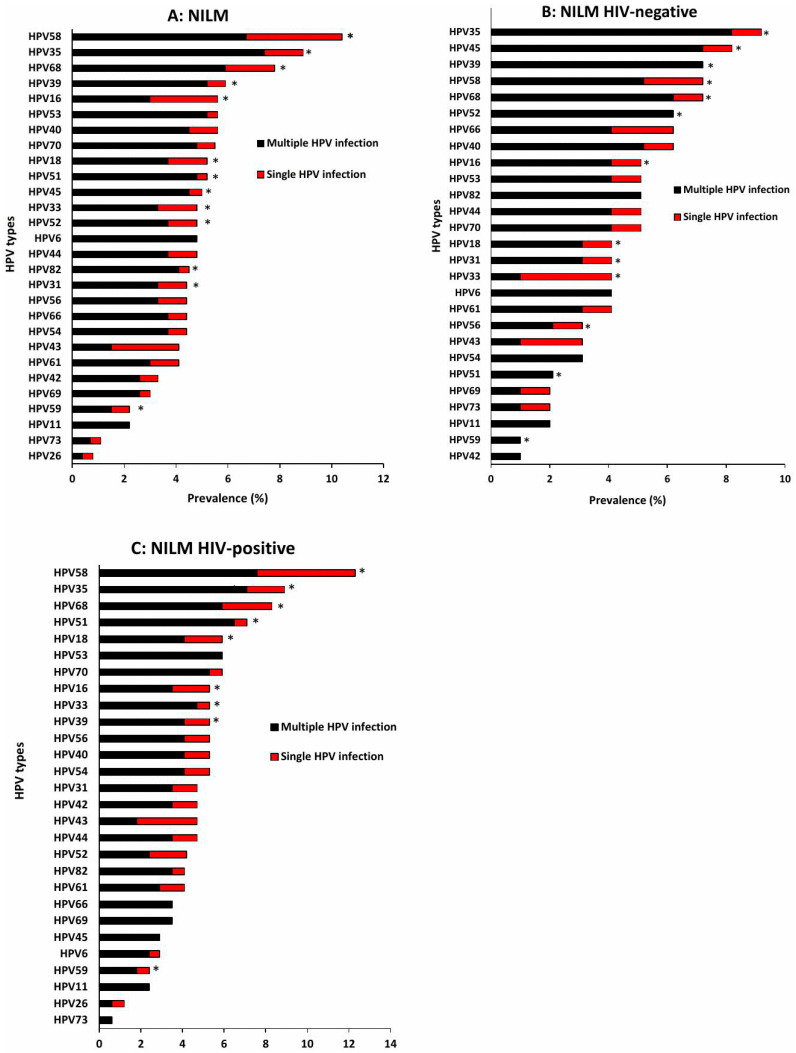
Distribution of HPV genotypes among Eastern Cape women with normal cervical cytology, all women (**A**), HIV-negative (**B**) and HIV-positive women (**C**). NILM: Negative for Intraepithelial Lesion or Malignancy, * indicate high-risk HPV types.

**Figure 4:**
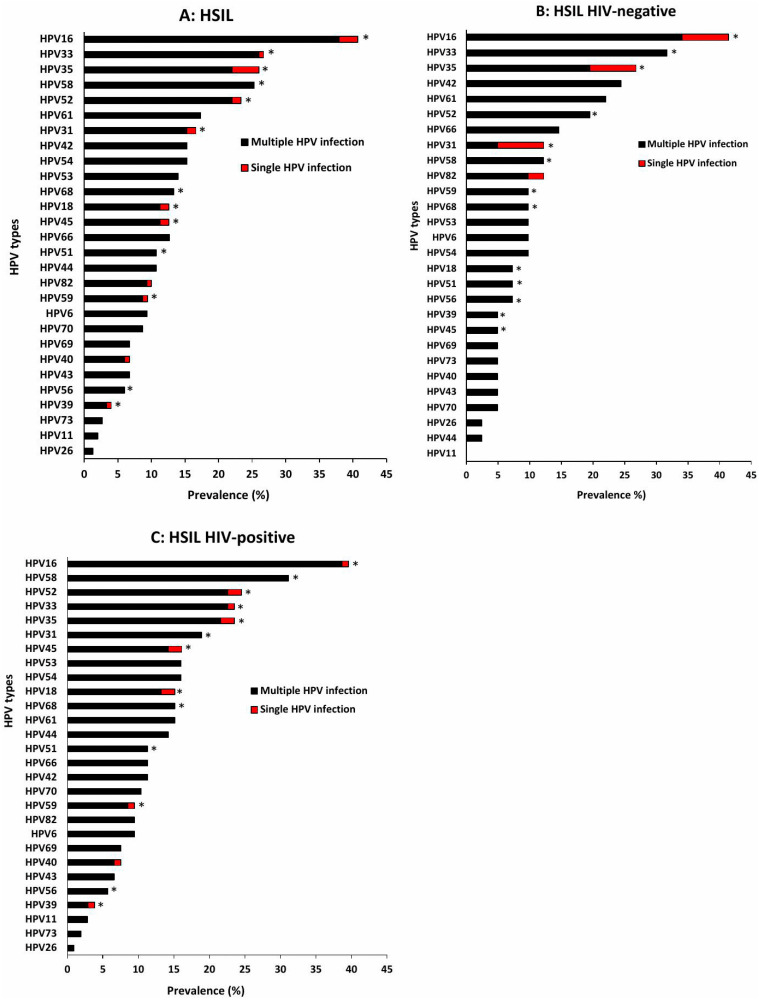
Distribution of HPV genotypes among Eastern Cape women with HSIL, all women (**A**), HIV-negative (**B**) and HIV-positive women (**C**). HSIL: High-grade Squamous Intraepithelial Lesion, * indicate high-risk HPV types.

Among women with NILM cytology, the five most prevalent HPV types overall were HPV58 (10.4%), HPV35 (8.9%), HPV68 (7.8%), HPV39 (5.9%), and HPV16 and 53 (5.6%). For most types, multiple infections occurred more frequently than single infections (Figure 3A). When stratified by HIV status, HPV35 (9.3%), HPV45 (8.2%), and HPV39, 58, and 68 (7.2%) were the most common HPV types among HIV-negative, with multiple infections again exceeding single infections (Figure 3B). In contrast, HIV-positive women showed higher prevalence of HPV58 (12.4%), HPV35 (8.8%), and HPV68 (8.2%), and similarly demonstrate a predominance of multiple over single HPV infections (Figure 3C). Among women with ASC-US cytology, HPV68 was the most dominant type, and multiple infections were more common than single infections. In LSIL, HPV16 predominated, with the same pattern of multiple infections being more frequent. ASC-H cases were also dominated by HPV16, with multiple infections more common overall; however, HPV16 was the most frequent single-type infection specifically among HIV-negative women. In HSIL, HPV16 (40.7%), HPV33 (26.7%), HPV35 (26.0%), HPV58 (25.3%), and HPV52 (23.3%) were the most prevalent HPV types, and all were detected more frequently in multiple infections than as single types (Figure 4A). Among HIV-negative HSIL cases, HPV16 (41.5%), HPV33 (31.7%), and HPV35 (26.8%) were the dominant types, with multiple infections again more common (Figure 4B). Among HIV-positive HSIL cases, HPV16 (39.6%), HPV58 (31.1%), and HPV52 (24.5%) were most prevalent, reflecting the same pattern of higher multiple-infection burden in HIV-positive women (Figure 4C).

**Figure 5:**
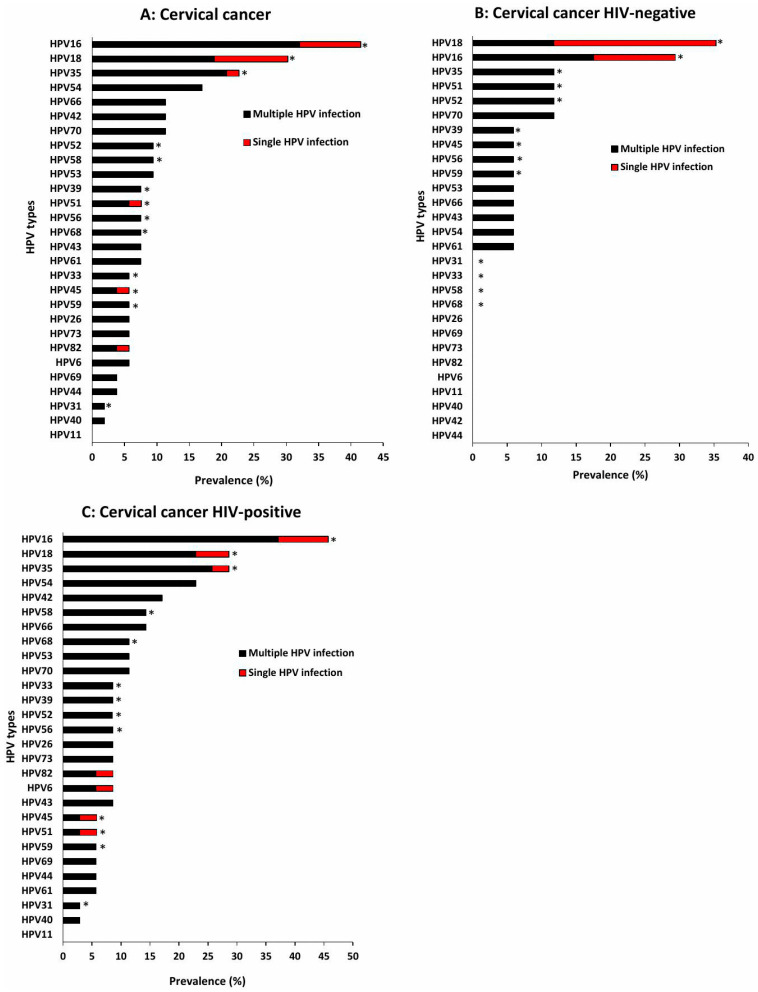
Distribution of HPV genotypes among Eastern Cape women with cervical cancer, all women (**A**), HIV-negative (**B**) and HIV-positive women (**C**). * indicate high-risk HPV types.

In contrast, among cervical cancer cases, the most prevalent HPV types overall were HPV16 (41.5%), HPV18 (30.2%), HPV35 (22.6%), HPV54 (17.0%), and HPV66 (11.3%). Multiple infections remained more common than single-type infections across all HPV types (Figure 5A). Among HIV-negative women with cervical cancer, the most dominant HPV types were HPV18 (35.3%), HPV16 (29.4%), and HPV35, 51, 52, and 70 (11.8%). Although multiple infections were still more frequent than single infections, HPV18 appeared more commonly as a single-type infection (Figure 5B). Among HIV-positive women, HPV16 (45.7%) was the most prevalent type, followed by HPV18 and 35 (28.6%), and HPV54 (22.9%). Consistent with patterns observed in other cytology grades, multiple infections remained more common than single infections (Figure 5C).

### 3.4. Prevalence of HPV Types Targeted by Current Commercial HPV Vaccines According to Different Cervical Cytology Grades

**Figure 6:**
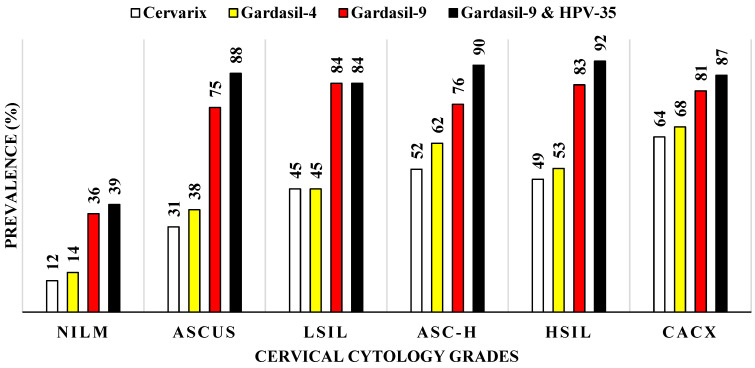
Prevalence of human papillomavirus types targeted by current commercial HPV vaccines according to cervical cytology grades among women of Eastern Cape Province, South Africa. (NILM: Negative for intraepithelial lesion or malignancy, ASC-US: Atypical squamous cells of undetermined significance, LSIL: Low-grade squamous intraepithelial lesion, ASC-H: Atypical squamous cells, cannot exclude high-grade squamous intraepithelial lesion, HSIL: High-grade squamous intraepithelial lesion, CACX: Cervical cancer, Cervarix^®^ vaccine target HPV16/18; Gardasil-4^®^ vaccine targets HPV6/11/16/18, and Gardasil-9^®^ vaccine targets HPV6/11/16/18/31/33/45/52/58).

HPV types targeted by Cervarix^®^ HPV vaccine were detected in 12.0% (31/269) of NILM cases, 31.0% (5/16) of ASC-US, 45.0% (14/31) of LSIL, 52.0% (11/21) of ASC-H, 49.0% (73/150) of HSIL, and 64.0% (34/53) of cervical cancer cases. HPV types targeted by Gardasil-4^®^ HPV vaccine were detected in 14.0% (39/269) of NILM, 38.0% (6/16) of ASC-US, 45.0% (14/31) of LSIL, 62.0% (13/21) of ASC-H, 53.0% (79/150) of HSIL, and 68.0% (36/53) of cervical cancer cases. HPV types targeted by Gardasil-9^®^ HPV vaccine were detected in 36.0% (97/269) of NILM, 75.0% (12/16) of ASC-US, 84.0% (26/31) of LSIL, 76.0% (16/21) of ASC-H, 83.0% (125/150) of HSIL, and 81.0% (43/53) of cervical cancer cases. When HPV35 was added to the Gardasil-9^®^ HPV vaccine, prevalence increased to 39.0% (106/269) for NILM, 88.0% (14/16) for ASC-US, 84.0% (26/31) for LSIL, 90.0% (19/21) for ASC-H, 92.0% (138/150) for HSIL, and 87.0% (46/53) for cervical cancer. Notably, no additional increase was observed in LSIL, where the proportion remained unchanged following the inclusion of HPV35 (Figure 6). HPV35 was included due to its disproportionately high prevalence among African women, despite not being covered by existing vaccines. Incorporating HPV35 may therefore help close an important protection gap for populations with a higher HPV35-associated cervical cancer burden.

### 3.5. Factors Associated with HPV Infection Among Women with Normal Cervical Cytology in the Eastern Cape Province

Among women with normal cervical cytology, age was strongly associated with HPV infection. Participants aged 18–29 years had the highest prevalence, which declined significantly among those aged 40–49 years and 50–60 years (RR: 0.59, 95% CI: 0.40–0.81, *p* = 0.001; RR: 0.61, 95% CI: 0.45–0.80, *p* < 0.001, respectively). Current or past alcohol consumption was associated with a higher prevalence of HPV infection compared with non-drinkers (RR: 1.43, *p* = 0.029 and RR: 1.44, *p* = 0.021, respectively). Past smokers were also at significantly higher risk than non-smokers (RR: 1.43, 95% CI: 1.09–1.71, *p* = 0.020). HPV prevalence was higher among single women compared with those who were married or cohabiting (RR: 0.68, 95% CI:0.49–0.89, *p* = 0.004) and those who were separated or widowed (RR:0.34, 95% CI: 0.12–0.75, *p* = 0.002). Having one sexual partner in the past year compared to no partner was associated with increased HPV prevalence (RR: 1.39, 95% CI: 1.04–1.98, *p* = 0.031, Table 3).

Women with five or more children had significantly lower HPV infection risk (RR: 0.45, 95% CI: 0.29–0.70, *p* = 0.002). Use of condoms as their primary contraceptive method was associated with higher HPV prevalence compared to no contraceptive use (RR: 1.43, 95% CI: 1.05–1.87, *p* = 0.028). Participants reporting vaginal discharge also had a significantly higher prevalence of HPV infection (RR: 1.40, 95% CI: 1.15–1.72, *p* = 0.0001). Early sexual debut, HIV status, level of education, and other investigated factors were not significantly associated with HPV infection (*p* > 0.05). Among women with HSIL and cervical cancer, none of the assessed behavioral or demographic factors were significantly associated with HPV infection except for HIV status. HIV-positive women had an increased risk of HPV infection compared to HIV-negative women (RR: 1.09, CI: 1.02–1.24, *p* = 0.019, Table 3).

## 4. Discussion

This study examined the distribution of HPV genotypes and associated risk factors among women in the Eastern Cape Province across varying cervical cytology grades, with additional stratification by HIV status. Women with high-grade lesions or cervical cancer tended to be older, whereas those with normal cytology were generally younger. The progressive increase in median age with worsening cytological abnormalities suggest that cervical cancer development is more strongly linked to chronic, persistent HPV infection rather than recent exposure [29]. The age differences observed between HIV-negative and HIV-positive women further indicate that HIV-related immune impairment may reduce HPV clearance, thereby increasing viral persistence [25,30]. HPV prevalence was high across all cervical cytology grades, consistent with findings from other studies reporting elevated HPV infection rates; particularly in women with HSIL and cervical cancer [10,31,32]. However, a study from KwaZulu-Natal, South Africa, reported lower HPV prevalence across these grades [33]. Collectively, these findings reinforced the central role of HPV in cervical carcinogenesis.

Multiple infections were more common among women with severe lesions and cervical cancer, aligning with earlier studies demonstrating that multiple infections are associated with higher viral loads and increased risk of progression [34,35,36]. This pattern underscores the complexity of HPV epidemiology in this high-risk population. Similarly to reports from other African settings, high-risk HPV types predominated across the cytological spectrum [36,37], while low-risk types were frequently detected in combination with high-risk types, particularly among HIV-positive women. This co-occurrence has been documented in other African cohorts, where high-risk and low-risk types commonly coexist within the same infections [38].

Genotype-specific analyses showed that women with normal cytology were most frequently infected with HPV58, 35, 68, 39, and 53, whereas women with HSIL were more likely to have HPV16, 33, 35, 58, and 52. These patterns align with previous studies that similarly reported HPV58 as one of the dominant types in women with normal cervical cytology [39,40] and HPV16 as the predominant type among women with HSIL [32,36,37]. In cervical cancer cases, HPV16 and HPV18 remained the most prevalent types, consistent with global evidence identifying these two genotypes as the primary oncogenic drivers of cervical cancer [41,42]. Notably, HPV35 was also common in cervical cancer, ranking immediately after HPV16 and HPV18, underscoring its epidemiological importance in Africa. The recurrent detection of HPV35 across all cytology categories underscores the need to consider expanding vaccine coverage to include this type, which is not protected by current Gardasil-9^®^ vaccine [15].

HIV-positive women showed a wide variety of HPV types and multiple infections, confirming that immune suppression facilitates persistent infections and viral spread [43], and emphasizing the importance of integrated HPV and HIV management in affected populations [43]. Interestingly, HPV54, a low-risk type, appeared in some cervical cancers among HIV-positive women, suggesting that certain low-risk types may behave differently in the context of HIV co-infection. Cervarix^®^ and Gardasil-4^®^ prevented only 12.0–14.0% of infections in women with normal cervical cytology but prevented 60.0% of infections in cervical cancer cases, indicating vaccine-targeted high-risk types primarily drive severe disease. Approximately 80.0% of infections in women with HSIL and cervical cancer were preventable through Gardasil-9^®^’s broader coverage. Protection increased further when HPV35 was included in the vaccine. This highlights the significance of HPV35 in Africa and demonstrates why its inclusion in future vaccines would be beneficial [15].

Behavioral factors associated with HPV infection in women with normal cervical cytology included being younger, single, a former smoker, an alcohol consumer, and reporting vaginal discharge. These findings are consistent with other African studies that have linked such factors to increased HPV risk [44,45]. The association between HPV and vaginal discharge may reflect recently acquired HPV infection occurring alongside other genital infections that cause inflammation and mucosal disruption, thereby facilitating HPV transmission [46,47]. In contrast to studies reporting high parity as a risk for HPV infection [48,49], women with more children in this study had a lower prevalence of HPV infection. This may be explained by the tendency for higher-parity women to be older, as HPV acquisition generally decreases with age. Condom users were more likely to contract HPV, likely due to inconsistent [50] and higher rates of other risk factors, such as earlier sexual debut and multiple partners. The observed association between reporting one sexual partner in the past year and increased HPV prevalence is likely due to cumulative lifetime exposure and viral persistence rather than recent acquisition. HPV prevalence is strongly influenced by prior sexual history, and persistent HPV DNA may still be detected even when recent new partners are excluded in cross-sectional studies [51]. HIV infection was a significant risk factor among women with HSIL and cervical cancer [26], but it was not linked to HPV infection in women with normal cervical cytology.

The study’s HPV genotyping, distinguishing single and multiple infections, provides detailed insight into HPV genotype distribution, including high-risk and probable high-risk types, beyond the most prevalent strains. Stratification by cervical cytology and HIV status enabled assessment of the impact of immune status on HPV diversity and disease severity, which is particularly relevant in high HIV prevalence settings such as the Eastern Cape. The relatively large sample size supports robust statistical analyses and enhances the representativeness of the findings. However, the cross-sectional design limits causal links regarding HPV persistence or progression. The small sample size of cervical cancer, ASC-US, LSIL and ASC-H cases restricted subgroup analyses and limits generalizability. Although HPV genotype coverage relative to vaccine types was evaluated, actual vaccination uptake, history, and effectiveness in the cohort, particularly since public vaccination began in 2014 targeting girls now aged 19–22 years, were not assessed, which may influence genotype prevalence. Participants’ prior treatment history for cervical lesions or cervical cancer was not collected, which could potentially influence HPV prevalence estimates, and cervical lesion diagnoses were derived from routine clinical reports and were not independently morphologically verified, which may introduce diagnostic variability. Additionally, analyses of self-reported behavioral variables, such as condom use and sexual history, may be subject to recall or social desirability bias, potentially affecting accuracy.

## 5. Conclusions

This study highlights the high overall HPV prevalence, which further increased among women with abnormal cervical cytology in the Eastern Cape Province, South Africa. While overall HPV prevalence was not strongly influenced by HIV co-infection, distinct differences were observed in the HPV genotype patterns when stratified by HIV status. A significant proportion of HSIL and cervical cancer cases were attributed to types covered by the Gardasil-9^®^, but the dominance of non-vaccine types, especially HPV35, underscores a critical gap in current prevention strategies relevant to this specific population. The substantial increase in potential vaccine coverage achieved by including HPV35 highlights the necessity of regionally tailored vaccine formulations or expanded coverage to effectively reduce the burden of cervical cancer in the Eastern Cape. Data generated from this study further encourages the use of the Gardasil-9^®^ HPV vaccine in the South African national screening programme. In evaluating regional HPV epidemiology, integrated strategies that combine HPV vaccination, HIV care, and targeted cervical cancer screening should be prioritized in regional prevention programs. Public health education should also address behavioral risk factors to reduce the acquisition of HPV. Larger-scale, longitudinal studies are essential for monitoring the effects of HPV vaccination, assessing HPV persistence, and informing regional vaccine policy and cervical cancer prevention initiatives.

## Figures and Tables

**Table 1:** Demographic characteristics of study participants.

Characteristics	NILM		ASC-US		LSIL		ASC-H		HSIL		Cervical Cancer	
	*n*/N	%	*n*/N	%	*n*/N	%	*n*/N	%	*n*/N	%	*n*/N	%
Age **^∑^**												
18–29 years	74/268	27.6	3/16	18.8	10/31	32.3	2/21	9.5	4/150	2.7	1/53	1.9
30–39 years	84/268	31.3	4/16	25.0	7/31	22.6	6/21	28.6	37/150	24.7	7/53	13.2
40–49 years	68/268	25.4	7/16	43.8	10/31	32.3	10/21	47.6	55/150	36.7	13/53	24.5
50–98 years	42/268	15.7	2/16	12.5	4/31	12.9	3/21	14.3	54/150	36.0	32/53	60.4
Marital status **^∑^**												
Single	195/267	73.0	13/16	81.3	26/31	83.9	10/21	47.6	81/148	54.7	26/52	50.0
Married	53/267	19.9	1/16	6.3	5/31	16.1	9/21	42.9	46/148	31.1	20/52	38.5
Widowed & Separated	19/267	7.1	2/16	12.5	0/31	0.0	2/21	9.5	21/148	14.2	6/52	11.5
Education **^∑^**												
None	0/267	0.0	1/16	6.3	1/31	3.2	1/21	4.8	9/149	6.0	4/52	7.7
Primary	17/267	6.4	2/16	12.5	1/31	3.2	3/21	14.3	36/149	24.2	17/52	32.7
Secondary	141/267	52.8	7/16	43.8	20/31	64.5	10/21	47.6	61/149	40.9	23/52	44.2
Tertiary	109/267	40.8	6/16	37.5	9/31	29.0	7/21	33.3	43/149	28.9	8/52	15.4
Currently working **^∑^**												
Yes	104/267	39.0	2/16	12.5	8/31	25.8	2/21	9.5	30/149	20.1	3/52	5.8
No	163/267	61.0	14/16	87.5	23/31	74.2	19/21	90.5	119/149	79.9	49/52	94.2
Currently Smoking **^∑^**												
Yes	30/267	11.2	4/16	25.0	9/31	29.0	7/21	33.3	20/148	13.5	7/52	13.5
No	210/267	78.7	8/16	50.0	8/31	25.8	5/21	23.8	116/148	78.4	42/52	80.8
Ex-smoker	27/267	10.1	4/16	25.0	14/31	45.2	9/21	42.9	12/148	8.1	3/52	5.8
Alcohol use **^∑^**												
Yes	41/267	15.4	0/16	0.0	7/31	22.6	4/21	19.0	9/149	6.0	3/51	5.9
No	111/267	41.6	8/16	50.0	11/31	35.5	12/21	57.1	104/149	69.8	40/51	78.4
Sometimes	70/267	26.2	7/16	43.8	11/31	35.5	2/21	9.5	17/149	11.4	3/51	5.9
Past	45/267	16.9	1/16	6.3	2/31	6.5	3/21	14.3	19/149	12.8	5/51	9.8
Sexual debut age **^∑^**												
≤16 years	57/267	21.3	4/16	25.0	12/31	38.7	7/21	33.3	55/146	37.7	13/52	25.0
17–18 years	105/267	39.3	4/16	25.0	7/31	22.6	5/21	23.8	46/146	31.5	18/52	34.6
19–20 years	56/267	21.0	2/16	12.5	5/31	16.1	4/21	19.0	27/146	18.5	12/52	23.1
≥21 years	49/267	18.4	6/16	37.5	7/31	22.6	5/21	23.8	18/146	12.3	9/52	17.3
Once Pregnant **^∑^**												
Yes	226/266	85.0	15/16	93.8	28/31	90.3	21/21	100.0	145/148	98.0	52/52	100.0
No	40/266	15.0	1/16	6.3	3/31	9.7	0/21	0.0	3/148	2.0	0/52	0.0
HIV status **^∑^**												
Negative	97/267	36.3	6/16	37.5	8/31	25.8	4/21	19.0	41/147	27.9	17/52	32.7
Positive	170/267	63.7	10/16	62.5	23/31	74.2	17/21	81.0	106/147	72.1	35/52	67.3
Number of lifetime sexual partners **^∑^**												
1	17/267	6.4	1/16	6.3	2/31	6.5	2/21	9.5	20/146	13.7	5/52	9.6
2	39/267	14.0	4/16	25.0	7/31	22.6	4/21	19.0	29/146	19.9	14/52	26.9
3–4	117/267	43.8	7/16	43.8	12/31	38.7	6/21	28.6	52/146	35.6	23/52	44.2
≥5	94/267	35.2	4/16	25.0	10/31	32.3	9/21	42.9	45/146	30.8	10/52	19.2

**^∑^**: Indicate the number of participants with available data for each variable. Percentages were calculated based on available data; missing values/non-respondents are not included. NILM: Negative for Intraepithelial Lesion or Malignancy, ASC-US: Atypical Squamous Cells of Undetermined Significance, LSILs: Low-grade Squamous Intraepithelial Lesions, ASC-H: Atypical Squamous Cells cannot exclude High-grade Lesion, HSILs: High-grade Squamous Intraepithelial Lesions.

**Table 2:** HPV prevalence according to cervical cytology among women of the Eastern Cape, South Africa.

Variables	Normal	ASC-US	LSIL	ASC-H	HSIL	CaCx
	% (*n*/269)	% (*n*/16)	% (*n*/31)	% (*n*/21)	% (*n*/150)	% (*n*/53)
Any type	60.6 (163)	93.8 (15)	100.0 (31)	95.2 (20)	93.7 (145)	92.5 (49)
Single infection	29.7 (80)	12.5 (2)	22.6 (7)	19.0 (4)	14.7 (22)	28.3 (15)
Multiple infection	30.9 (83)	81.3 (13)	77.4 (24)	76.2 (16)	80.7 (121)	64.2 (34)
HR-HPV	47.2 (127)	87.5 (14)	100.0 (31)	95.2 (20)	95.3 (143)	90.6 (48)
Probable HR-HPV	14.5 (39)	25.5 (4)	41.7 (13)	23.8 (5)	34.0 (51)	28.3 (15)
LR-HPV	27.9 (75)	37.5 (6)	54.8 (17)	52.4 (11)	52.0 (78)	35.8 (19)

ASC-US: Atypical Squamous Cells of Undetermined Significance, LSILs: Low-grade Squamous Intraepithelial Lesions, ASC-H: Atypical Squamous Cells cannot exclude High-grade Lesion, HSILs: High-grade Squamous Intraepithelial Lesions, CaCx: Cervical Cancer. HR-HPV types: HPV16, 18, 31, 33, 35, 39, 45, 51, 52, 56, 58, 59, and 68. Probable HR-HPV types: HPV26, 53, 66, 69, 73, and 82. LR-HPV types: HPV6, 11, 40, 42, 43, 44, 54, 61, and 70.

**Table 3:** Factors associated with HPV infection among women with normal cervical cytology in the Eastern Cape Province, South Africa.

Variable		HPV Prevalence %, *n*/N	RR (95% CI)	*p*-Value
Age				
	18–29 years	77.0%, 57/74	Reference	
	30–39 years	64.3%, 54/84	0.83 (0.68–1.02)	0.085
	40–49 years	47.1%, 32/68	0.61 (0.45–0.80)	**<0.0001**
	50–60 years	45.2%, 19/42	0.59 (0.40–0.81)	**0.0001**
Sexual debut age				
	≤16 years	61.4%, 35/57	Reference	
	17 years	66.7%, 30/45	1.09 (0.80–1.46)	0.680
	18 years	56.7%, 34/60	0.92 (0.68–1.25)	0.707
	19–20 years	50.0%, 28/56	0.81 (0.58–1.13)	0.258
	≥21 years	69.4%, 34/49	1.13 (0.85–1.50)	0.420
HIV status				
	Negative	56.7%, 55/97	Reference	
	Positive	62.4%, 106/170	1.10 (0.90–1.37)	0.367
Education				
	Primary	70.6%, 12/17	Reference	
	Secondary	55.3%, 78/141	0.78 (0.60–1.21)	0.303
	Tertiary	65.1%, 71/109	0.92 (0.71–1.42)	0.787
Ever consumed alcohol				
	No	47.7%, 53/111	Reference	
	Yes	68.3%, 28/41	1.43 (1.05–1.88)	**0.029**
	Sometimes	70.1%, 49/70	1.47 (1.14–1.88)	**0.004**
	Past	68.9%, 31/45	1.44 (1.08–1.88)	**0.021**
Smoker				
	No	57.1%, 120/210	Reference	
	Yes	63.3%, 19/30	1.11 (0.78–1.42)	0.559
	Past	81.5%, 22/27	1.43 (1.09–1.71)	**0.020**
Currently working				
	No	62.0%, 101/163	Reference	
	Yes	57.7%, 60/104	0.93 (0.75–1.14)	0.522
Marital status				
	Single	67.2%, 131/195	Reference	
	Married/cohabiting	45.8%, 27/59	0.68 (0.49–0.89)	**0.004**
	Separated/widowed	23.1%, 3/13	0.34 (0.12–0.75)	**0.002**
Number of lifetime sexual partners				
	1–2	55.4%, 31/56	Reference	
	3	62.2%, 46/74	1.12 (0.84–1.53)	0.474
	4	65.1%, 28/43	1.18 (0.84–1.63)	0.410
	≥5	59.6%, 56/94	1.08 (0.82–1.46)	0.733
Number of sexual partners in the past year				
	0	45.8%, 22/48	Reference	
	1	63.6%, 110/173	1.39 (1.04–1.98)	**0.031**
	2–4	63.0%, 29/46	1.38 (0.95–2.04)	0.103
Number of sexual partners in the past month				
	0	55.8%, 43/77	Reference	
	1–2	62.1%, 118/190	1.11 (0.90–1.42)	0.408
Condom use during last sexual intercourse				
	No	62.9%, 105/167	Reference	
	Yes	55.6%, 55/99	0.88 (0.71–1.08)	0.247
	I don’t remember	100.0%, 1/1	1.59 (0.33–3.57)	1.000
Ever pregnant				
	No	72.5%, 29/40	Reference	
	Yes	58.0%, 131/226	0.80 (0.66–1.04)	0.114
Parity				
	0	87.5%, 14/16	Reference	
	1	66.7%, 40/60	0.76 (0.60–1.08)	0.129
	2	60.7%, 34/56	0.68 (0.53–0.99)	0.070
	3–4	62.1%, 41/66	0.71 (0.55–1.01)	0.075
	≥5	39.4%, 15/38	0.45 (0.29–0.70)	**0.002**
	No response	54.8%, 17/31	0.63 (0.42–0.93)	**0.049**
Contraceptives				
	None	51.6%, 49/95	Reference	
	Pills	50.0%, 7/14	0.97 (0.51–1.52)	1.000
	Condoms	73.5%, 25/34	1.43 (1.05–1.87)	**0.028**
	Intrauterine device	66.7%, 8/12	1.29 (0.74–1.84)	0.373
	Depoprovera	64.7%, 55/85	1.26 (0.98–1.62)	0.096
	Nur-Isterate	69.2%, 9/13	1.34 (0.80–1.87)	0.255
	Withdrawal or other	53.8%, 7/13	1.04 (0.55–1.60)	1.000
Vaginal discharge				
	No	50.4%, 68/135	Reference	
	Yes	70.5%, 93/132	1.40 (1.15–1.72)	**0.0001**
Genital warts/blisters				
	No	59.6%, 84/141	Reference	
	Yes	61.1%, 77/126	1.03 (0.84–1.25)	0.804

The *p*-value bolded indicates significance. RR: Risk ratios.

## Data Availability

The data presented in this study are available on request from the corresponding author.

## Abbreviations

The following abbreviations are used in this manuscript:

HPV	Human papillomavirus
HIV	Human Immunodeficiency Virus
ASC-US	Atypical squamous cells of undetermined significance
LSILs	Low-grade intraepithelial lesions
ASC-Hs	Atypical squamous cells cannot exclude high-grade squamous intraepithelial lesions
HSILs	High-grade squamous intraepithelial lesions
HR-HPV	High-risk human papillomavirus
LR-HPV	Low-risk human papillomavirus
ART	Antiretroviral therapy
HREC	Health Research Ethics Committee
KSD	King Sabata Dalindyebo Municipality
PCR	Polymerase Chain Reaction
IQR	Interquartile range
CI	Confidence Interval
NILM	Negative for intraepithelial lesions or malignancy
CACX	Cervical cancer
NMAH	Nelson Mandela Academic Hospital
